# How We Read Oncologic FDG PET/CT

**DOI:** 10.1186/s40644-016-0091-3

**Published:** 2016-10-18

**Authors:** Michael S. Hofman, Rodney J. Hicks

**Affiliations:** 1 Centre for Molecular Imaging, Dept of Cancer Imaging, Peter MacCallum Cancer Centre, 305 Grattan Street, Melbourne, 3000 Australia; 2 Sir Peter MacCallum Department of Oncology and Department of Medicine, University of Melbourne, Melbourne, Australia

**Keywords:** Fluorodeoxyglucose FDG, Positron-emission tomography, Radiology, Medical oncology

## Abstract

^18^F-fluorodeoxyglucose (FDG) PET/CT is a pivotal imaging modality for cancer imaging, assisting diagnosis, staging of patients with newly diagnosed malignancy, restaging following therapy and surveillance. Interpretation requires integration of the metabolic and anatomic findings provided by the PET and CT components which transcend the knowledge base isolated in the worlds of nuclear medicine and radiology, respectively. In the manuscript we detail our approach to reviewing and reporting a PET/CT study using the most commonly used radiotracer, FDG. This encompasses how we display, threshold intensity of images and sequence our review, which are essential for accurate interpretation. For interpretation, it is important to be aware of benign variants that demonstrate high glycolytic activity, and pathologic lesions which may not be FDG-avid, and understand the physiologic and biochemical basis of these findings. Whilst FDG PET/CT performs well in the conventional imaging paradigm of identifying, counting and measuring tumour extent, a key paradigm change is its ability to non-invasively measure glycolytic metabolism. Integrating this “metabolic signature” into interpretation enables improved accuracy and characterisation of disease providing important prognostic information that may confer a high management impact and enable better personalised patient care.

## Background

^18^F-fluorodeoxyglucose (FDG) PET/CT imaging has become a key modality for imaging patients with cancer [1]. The process of reviewing PET/CT studies involves integration of the metabolic findings from the FDG component combined with the anatomical information provided by the CT component. This is a modality with many patterns of structural, physiologic and biochemical abnormalities that transcend the boundaries previously isolated in the worlds of nuclear medicine or radiology in characterising pathological conditions, particularly including cancer. Whilst there is a wealth of literature addressing the utility of PET in a large array of malignancies, the art of how to review and interpret PET/CT is generally acquired like an apprentice and not well addressed in the literature. In this article, we detail our approach to reviewing a PET/CT study using the most commonly used tracer, FDG. Future articles in this series will address the use of other tracers pertinent to other cancers.

### Acquisition

Patient preparation is important in acquiring good quality studies and it is the responsibility of the PET specialist to ensure that appropriate protocols are in place to prevent non-diagnostic or suboptimal studies. Detailed discussion of acquisition parameters is beyond the scope of this review but includes preparation of diabetic patients, strategies to minimise brown fat activation, as well as prescription of the extent of the field-of-view and the positioning of the patient to address the clinical question. For example, we position the patient with their arms down for head and neck malignancies but with their arms up for thoracic cancers. It is also important to determine the methodology to be used for CT acquisition. This varies widely according to local practice and our approach is discussed in further detail later in this manuscript.

An important aspect of interpretation is assessment of the technical adequacy of the study and ideally should be done before the patient leaves the department to enable repeat acquisition of any critical regions inadequately assessed on the initial examination.

### Optimal windowing of PET images

In any PET/CT study there are three discrete image sets that require display. These are the stand-alone PET data, the CT and the fused PET/CT images. Correct and consistent windowing is key to avoid both over- and under-interpretation of findings and to maintain the consistency required for accurate comparison of multiple studies. This also aids presentation of findings to referrers and patients.

The primary data from PET has been traditionally displayed on a linear grey scale. This is because the human eye is adept at discerning subtle differences in contrast from white through grey to black. The lower threshold of this display should be set at zero (white) while the upper threshold needs to be manipulated to obtain consistent display of physiological and pathologic uptake. Consequently, the intensity of normal tissues should be within the lower-to-middle portion of the dynamic range while the upper range used to demonstrate the range of intensities that might exist in pathological processes characterised by high glycolytic activity. By maintaining a reasonable spectrum of grey shades for display of normal tissues it is possible to detect faint lesions in areas of low background activity, such as the lung.

**Fig. 1:**
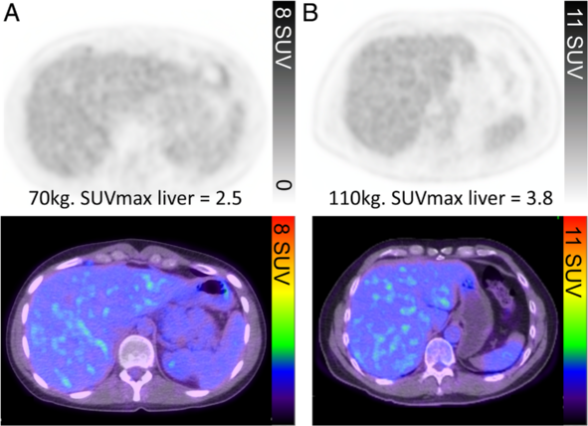
The PET window intensity is adjusted so that the liver appears light to mid-grey on the grey scale, corresponding to flecks of green in the liver on the rainbow colour scale. Despite the difference in SUVmax of the liver secondary to differences in weights of the two patients (**a** and **b**), the liver intensity this appears the same in both patients

Our preference is to have the most intense voxels in the normal liver appearing just below the middle of the grey scale range, which will be a light to mid-grey (Fig. 1a). Use of a colour scale is required for superimposition of functional images over the CT. We prefer to use the "rainblow" colour scale that has low activity regions displayed in the blue-green range and higher intensity regions in the orange-red spectrum. With this colour scale, the liver will generally appear blue with flecks of green with adjusgment if not (Fig. 1). This corresponds to an upper SUV window threshold of 8–10 and will usually achieve an appropriate contrast, except in very large patients in whom this may make the liver too dark. This is because adipose tissue contributes to the weight correction of administered activity, which is used for SUV calculation, but does not itself take up FDG. This means that more FDG is available for uptake in other tissues, including the liver. However, this may be counteracted by deposition of fat in the liver in obese subjects. This will usually be apparent by virtue of increased relative uptake in the spleen, which is generally marginally less intense than the liver. The brain will usually be nearly black with this scaling. This is unless cortical glycolytic activity is reduced by metabolic processes, especially by hyperglycaemia, or neurological conditions such as dementia. In children requiring general anaesthesia during the uptake and scanning procedure, cortical activity can also be significantly reduced. There are also changes in the brain during childhood maturation [2].

Under fasting conditions, glucose and its analogue, FDG, have facilitated uptake into the liver and therefore generally this organ has significantly higher activity than the blood. By definition, any structure with uptake more intense than that in the liver must also have facilitated FDG uptake and trapping. The advantage of using the liver as a reference tissue is also aided by this organ having rather low variability in metabolic activity [3]. It is, however, inappropriate to threshold for liver uptake if it is not deemed normal due to diffuse malignant infiltration, sarcoidosis, or fatty infiltration. This can be detected visually if there is marked discrepancy between liver and spleen intensity, although with sarcoidosis or lymphoma both can be increased. Our practice of thresholding the grey and colour scale to liver as detailed above results in similar image intensity to a fixed upper SUV threshold of 8 to 10. However, using the liver as a reference enables consistent windowing of images over a series of time-points within and between individuals and compensates for variations that might be caused by inaccuracies in SUV measurement between scans, issues related to dose calibration errors, extravasation of dose, different uptake periods or technical differences if rescanned on a different type of PET/CT device. When the liver is abnormal and cannot be used as a reference organ, we use the default SUV setting of an upper SUV threshold of 8. The same SUV threshold as that used for the whole body study should be applied when additional separate series are acquired (e.g. of the limbs) that do not encompass the liver.

**Fig. 2:**
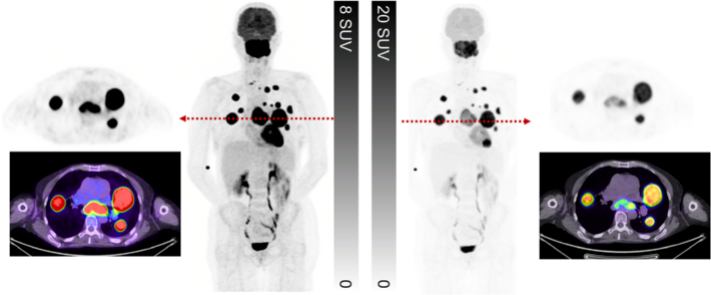
This patient presented with suspected metastatic nasopharyngeal cancer. Initial workup with endoscopic ultrasound and biopsy of the subcarinal node was non-diagnostic with necrotic tissue. FDG PET/CT demonstrates very intense uptake at all sites with lower uptake in the subcarinal node, only evident when widening the PET window. The findings suggest a different tumour biology at this site  with necrosis. When feasible, we recommend biopsy of the most FDG-avid lesion which likely represents the site of most aggressive disease and least likely to be non-diagnostic. In summary, the PET study windowed narrowly is primed for sensitivity whereas a wider window enables superior characterisation

Since some disease processes can have extremely high SUV values, it may be necessary to increase the upper threshold to appreciate the dynamic range of glycolytic activity. This is particularly important in diseases where there can be considerable heterogeneity in disease. Follicular lymphoma, in which most lesions can have a SUV_max_ in excess of 10 but regions of high-grade transformation with corresponding values of >15, is a particular case in point. Standard thresholds provide a good representation of the extent of disease but using a higher upper threshold to display the images can help to identify the regions of likely transformation or different disease biology and can aid biopsy site selection (Fig. 2).

This “rainbow” colour scale has relatively abrupt changes in colour, which enable easy differentiation of uptake intensity in the low, mid or high range. It is also a psychologically intuitive scheme with blue-green shades being cool colours whereas yellow-orange colours denote caution and reds, danger. Like a traffic light, we teach our referrers that these spectrums usually represent benign, equivocal and pathological findings, respectively. Clearly, this is an oversimplification, but it enables one to eyeball the PET image and decide if the uptake is of low, moderate or high metabolic activity.

**Fig. 3:**
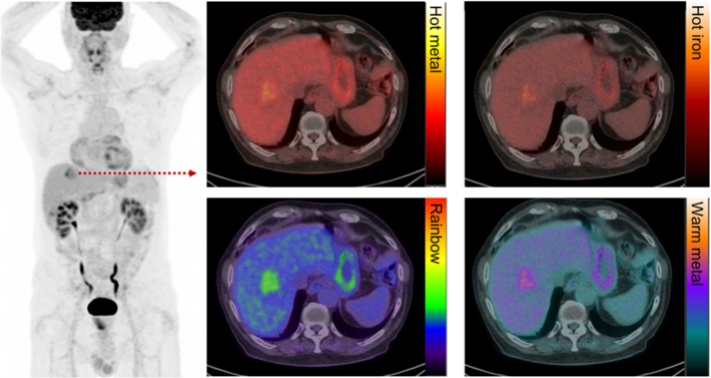
Patient with metastatic colorectal carcinoma and hepatic metastasis. The fused image is presented in different colour scales. We recommend using the “rainbow” scale owing to the superior tumour-to-liver contrast compared to other commonly used colour maps

It should, however, be noted that this can be a dangerous scale to use if there isn’t a disciplined and consistent use of the threshold setting principles detailed above since it is easy to “dial” lesions in and out. We often see studies, particularly from practices that have more experience with CT than PET, that have clearly had the threshold altered to render them red, or not, depending on whether the reader considers them more, or less, likely to be malignant based on the CT characteristics. While this might be a reasonable approach to communicate the site of a lesion, it diminishes the power of PET to characterise disease based on the degree of its metabolic activity. To avoid the risks associated with this scale, some manufacturers set the default colour scale to a dichotomous range, such as blue-yellow or brown-gold (see Fig. 3). This does not carry the psychological power of the rainbow scale but can be useful for displaying sites of presumed disease against the background of CT while reducing the risk of false-positive results due to use of an inappropriate display threshold. The “rainbow” colour scale may also be difficult for individuals with colour blindness to interpret.

We dislike colour scales with a continuous spectrum of a single colour, such as the commonly used “hot metal” scale, as these provide poor contrast between low and high intensity, and background CT images. The human eye is very sensitive in detecting differences of intensity within a grey scale but not so good within a single colour spectrum. Consequently, with “hot metal” or similar colour scales, it is difficult to qualitatively assess an image and know where the intensity of abnormality lies within in the spectrum. Moreover, the highest intensity on this scale is sometimes white, which is essentially uninterpretable when superimposed on a grey scale CT image.

Standardised windows have been developed that set upper and lower levels for Hounsfield units that optimally display the range of densities pertinent for a particular tissue. We routinely review soft tissue, lung and bone windows but in appropriate situations will use other specialised windows. Just as the profession has imposed certain discipline in the use of standardised windows for use on CT, we believe that there should be greater harmonisation of display of PET images.

### PET/CT review sequence

Initial review of the images blinded to patient history or indication is valuable as it enables an unbiased assessment. The black-and-white *cine* maximum intensity projection (MIP) is foremost in this initial review. This enables a “gestalt” impression of the study. The reconstruction method of these images tends to suppress noise and highlight regions of increased activity. Furthermore, the brain can appreciate these images as being volumetric, especially when rotating. This particularly aids recognition of the shape of regions increased activity, and particularly whether they are spherical, tubular or geographic. For the importance of this, see “Rod’s Rules” in the introduction to the “How We Read” series [4]. With experience, key findings are often established within seconds by review of this series. By definition, this image is relatively insensitive to regions of reduced activity.

**Fig. 4:**
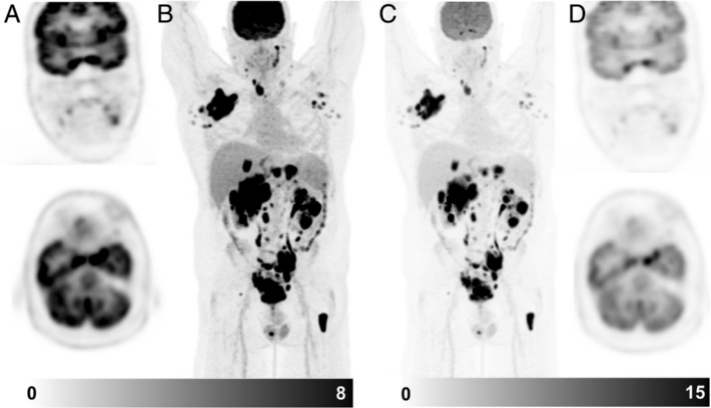
Patient with diffuse large B cell lymphoma. On the standard windowing, no abnormality is readily identified in the brain (**a** coronal & axial slice, **b** MIP image). By increasing the upper SUV threshold, abnormal uptake becomes readily becomes visible (**c** MIP image, **d** coronal & axial slice). This corresponded to a MRI abnormality which was not reported prospectively but identified following targeted review after the PET scan. Changing the PET window so that abnormalities can be identified above physiologic brain activity should be a routine component of image review

Next, we review the coronal PET images and triangulate apparent abnormalities on other planes and the MIP image. It is important to review these images on a workstation that has capacity to triangulate findings in axial, coronal and sagittal planes. We find the coronal images particularly helpful for detecting small abnormalities, particularly within the lungs and subcutaneous tissue. Any lesions identified on the PET are then correlated with the CT images, reviewing soft tissue, lung and bone windows as appropriate to the location of the abnormality. We selectively review the non-attenuation corrected (NAC) series when there is uncertainty about possible reconstruction artefacts due to metallic objects or patient movement between PET and CT components. Finally, it is important to widen the PET window in order to review the brain, otherwise easily discernible abnormalities can be missed (see Fig. 4).

Only after completing review of the stand-alone PET images we review the fused PET/CT images. This is a quite different process to that of many practices where the transaxial CT is scrolled through and any structural abnormalities identified are then correlated with the fused PET/CT image. This is often the preferred method of experienced radiologists who are sometimes more comfortable reviewing the CT than looking at stand-alone PET images. This approach tends to then use FDG information as an alternative contrast agent rather than as the primary data of a PET/CT study. Those disposed to this method will also generally prefer to obtain a full diagnostic CT as part of the examination. The advantages and disadvantages of these differing methods will be discussed subsequently.

As a final pass, we review the CT images sequentially on soft tissue, lung and bone windows to identify structural abnormalities not previously identified on PET review. Interpretation of structural abnormalities that are not associated with metabolic abnormality requires particular care and can give significant insights into the nature of pathological processes.

### Interpretation of PET/CT

The reader is directed to the initial article in this series, which details many of the principles that we use in formulating an impression of a scan, in reporting its findings and reaching a conclusion.

### Tumours grow as spheres: differentiating malignant from inflammatory aetiology

**Fig. 5:**
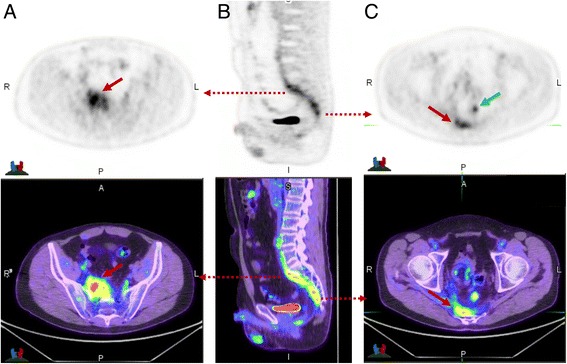
This patient had suspicion of pelvic recurrence in the setting of prior surgical excision for rectal carcinoma. There was intense uptake in the known pre-sacral soft tissue thickening (**a**) and (**c**) (red arrow) with SUVmax of 11. The linear morphology on the coronal image (**b**) suggested this was more likely inflammatory than malignant. A separate linear tract of metabolic activity was also seen (green arrow) extending from the pre-sacral abnormality to the peri-anal region (not shown). All abnormalities resolved following antiobiotic therapy confirming inflammatory aetiology

When high metabolic activity is present, one of the primary aims is to ascertain if the aetiology is malignant, benign or inflammatory. In early PET literature focusing on analysis of solitary pulmonary nodules, some researchers defined malignancy based on a SUV_max_ threshold of greater than 2.5 [5]. We contend that SUV analysis has virtually no role in this setting. Far more important than the SUV_max_ is the pattern rather than intensity of metabolic abnormality and the correlative CT findings. Our number one rule is that tumours grow as spheres, whereas inflammatory processes are typically linear and track along soft tissue boundaries such as pleural surfaces or fascial planes (see Fig. 5).

Occam's razor teaches us to look for a single cause that will explain all the findings on a particular study. One of the most challenging aspects of oncologic FDG PET/CT review, however, is to recognise all the patterns of metabolic activity that are not malignant and which consequently confound interpretation. Many benign and inflammatory processes are also associated with high glycolytic activity. Whilst some require further investigation, many have characteristic appearances that enable confident characterisation. A variety of potential pitfalls are detailed in Table 1, most of which do not require further investigation. Recognition of other pitfalls requires knowledge of the typical pattern of the various malignancies but is beyond the scope of this review. Future articles in the “How I Read” series will address the specific details of reading PET/CT in various cancers.

**Table 1:** Patterns of uptake in benign neoplasms, post treatment changes and inflammatory processes which can mimic malignancy

Pathology	Pattern of uptake and comment
Benign neoplasms	
Thyroid Hurthle cell adenoma (see Fig. 10a)	Focal and intense thyroid uptake. Virtually diagnostic if there is a calcified egg-shell appearance on CT, but this feature is not always present.
Renal oncocytoma (Fig. 10b)	Can have similar anatomic appearance on CT to renal cell carcinoma
Parotid oncocytoma (Warthin’s tumours) (see Fig. 10c)	Focal and intense uptake in the parotid corresponding to a soft tissue nodule of increased density relative to normal parotid tissue
Colorectal adenoma	These are typically focal and may be identified on CT if pedunculated. Depending on the clinical context, these generally warrant endoscopic evaluation as high FDG-avidity generally reflects at least high-grade dysplasia.
Elastofibromi dorsi	Linear low-to-moderate uptake corresponding to muscle-like soft tissue abnormality in posterior chest wall [17]
Post treatment changes	
Post talc pleurodesis (Fig. 6)	Multi-focal intense uptake corresponding to high-density material (talc) on CT. Can be extensive and persist indefinitely. FDG-avidity should be very closely matched to the sites of CT density and if performed for prior pleural malignancy, sites of pleural uptake with corresponding CT density should be considered to be malignant deposits
Post radiotherapy inflammatory change	Geographic (linear) change conforming to the radiation treatment field. Surrounding very low grade ‘haze’ of uptake within muscle and soft tissue can be apparent
Fat necrosis	Most commonly located in mesenteric fat after therapy in patients with lymphoma [10]. Focal moderate-to-intense uptake corresponding to nodule with density between that of soft tissue and fat. Classic appearance of ‘donut shape’ abnormality sometimes apparent.
High and symmetric tonsillar activity post chemotherapy	Commonly seen in patients with haematologic malignancies following chemotherapy, reflecting lymphoid repopulation/hyperplasia. This is commonly accompanied by lower grade reactive jugulodiagastric nodal activity that should not be misinterpreted as recurrent lymphoma.
Appendiceal linear activity	Another region of rich lymphoid tissue, in which increased activity is seen post treatment, particularly in younger patients as described above.
Dystrophic calcification	Following treatment some tumours calcify with pathologic correlate of xanthogranulomatosis inflammatory change. High metabolic activity can predate appearance of calcification on CT. Myositis ossificians is a variant of this process and should be considered for fusiform and focal intramuscular lesions, even in the absence of calcification. This can be an important diagnosis as this disease can mimic a sarcoma on MRI and even pathology.
Immune related inflammatory response	Following treatment with anti-CTLA4 antibodies (eg. iplilumab) and much less commonly with PD1 inhibitors (eg. pembrozulimab) low-to-moderate uptake in lymph nodes in drainage sites from tumours can be observed. Associated homogenous diffuse splenic uptake can assist identify this pattern. Autoimmune thyroiditis, colitis, adrenalitis and hypohysitis can also be identified in this therapeutic setting.
Inflammatory processes	
Hilar and mediastinal nodal activity, pre-caval nodal activity (see Fig. 7)	A common finding with symmetry being the key finding pointing to an inflammatory/reactive aetiology. Symmetrical nodal activity of malignant aetiology is exceedingly rare. We have noted higher incidence of this reactive pattern in patients from rural areas. In association, it is quite frequent to visualise similar intensity metabolic abnormality in the subdiaphragmatic pre-caval region.
Marrow uptake	Diffuse marrow uptake is a feature of a systemic inflammatory system and can be a feature of an infectious or septic process. It may be accompanied by mild diffuse increased splenic activity. This is also seen with Hodgkin’s lymphoma where only focal high intensity abnormalities should be interpreted as marrow infiltration.
Physiologic variants	
Anal sphincter activity	Midline, ring morphology, air-filled rectum (“polite sign”)
Fallopian tube and ovary (see Fig. 8)	In mid-cycle it is frequent to observe bilateral curvilinear increased fallopian tube activity +/− focal unilateral ovarian follicular activity [18]
Brown fat activity	Whilst typical features of symmetric cervical, supraclavicular, axillary and para-vertebral fat activity is easily identified, locations such as para-adrenal region should also be recognised. Administration of propranolol 10–20 mg orally 60 minutes prior FDG administration is effective in suppressing brown fat. Rarely, brown fat activation can be a clue to an underlying functional phaeochromocytoma or paraganglioma. Diffuse increased white fat uptake can also occur following administration of steroids [19].
Large and small bowel activity	Diffuse increased uptake is seen in patients on metformin, which increases colonic glycolysis. Cessation of metformin for 48 h will reduce bowel related activity [20]; this can be a useful manoeuvre if is interfering with scan interpretation. In patients not on metformin, physiologic bowel activity can be seen as part of normal peristalsis.
Ureteric activity	The ureter can follow a tortuous course which can result in apparent focal activity which can be difficult to distinguish from nodal activity. A delayed phase image after injection of intravenous contrast can assist by enabling confident localisation to the ureter.
Gallbladder luminal activity	Uncommon finding but seen in patients with a delayed uptake phase who have eaten after initial uptake period; this typically occurs when there is equipment failure necessitating very delayed imaging [21].
FDG ‘pulmonary emboli’	Iatrogenic micro-embolism can occur when blood is withdrawn from vein and mixed with FDG, and then re-injected. The complete absence of anatomic abnormality corresponding to focal very intense activity (SUV > 30) is very likely to represent this phenomenon.

**Fig. 6:**
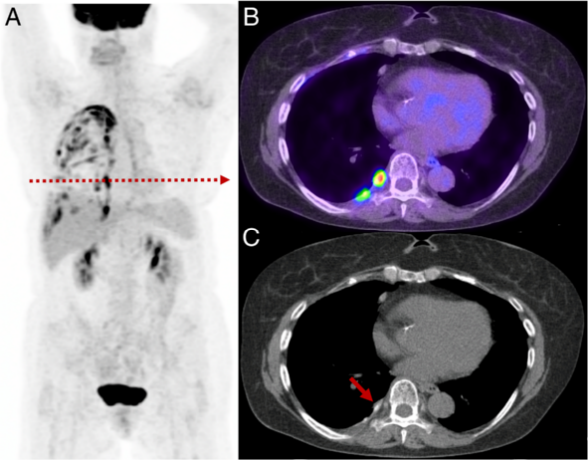
Patient with prior lung malignancy presents for surveillance. The study demonstrates a typical appearance of inflammatory change post talc pleurodesis with intense multi-focal uptake evident throughout the pleural surface (**a**). On the axial PET/CT (**b**) and CT (**c**) the high focal uptake correlates with a site of talc on CT recognised by its high density. Such change can persistent for many years after pleurodesis

**Fig. 7:**
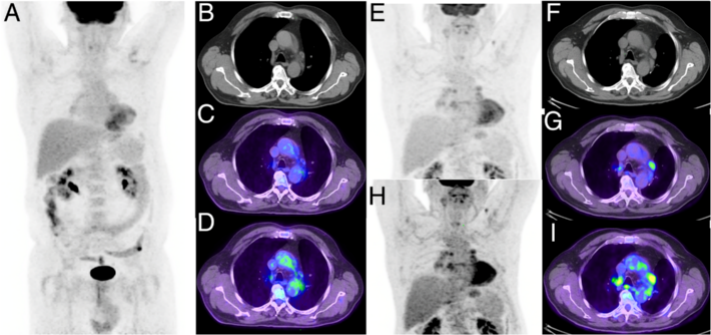
Patient with non-small cell lung cancer treated with curative intent radiotherapy. Post treatment restaging PET/CT demonstrated a complete metabolic response (**a**–**d**, **c** upper SUV threshold adjusted to liver background as detailed above, **d** upper SUV threshold of 5). Follow-up CT 9 months later demonstrated enlargement of multiple mediastinal nodes considered likely to represent malignant aetiology. Repeat PET/CT (**e**–**i**) demonstrated low-to-moderate uptake in these nodes. Given the symmetry of distribution in hilar and mediastinal nodes the aetiology was considered inflammatory, which was confirmed by resolution on follow-up. Thresholding the PET with a SUV threshold of 5 (**h**–**i**) might lead to erroneous description of intense uptake and interpretation as malignant in aetiology

**Fig. 8:**
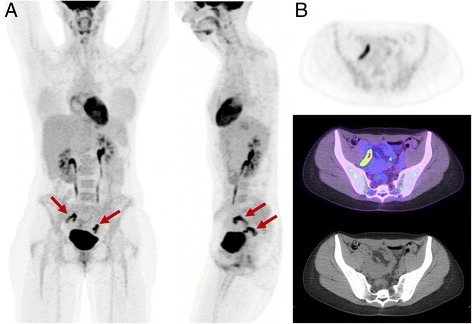
Appearance of physiologic adnexal uptake observed mid-cycle. Although the metabolic activity is high, on the rotating MIP images (**a** anterior and lateral) the activity is bilateral and curvilinear, characteristic of fallopian tube activity (**b**). Unilateral focal ovarian follicular activity is frequently seen in association with this finding

### Commonality of “Metabolic Signature”

**Fig. 9:**
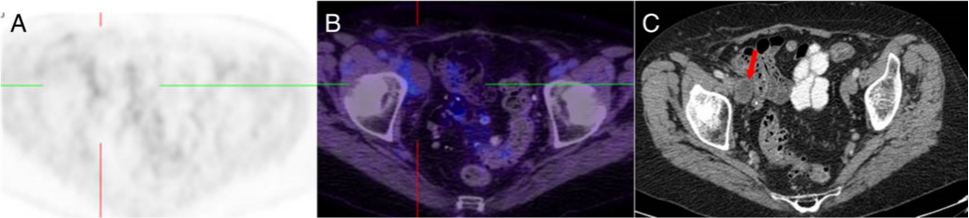
Patient with HPV-p16 positive cervical squamous cell carcinoma presents for staging. FDG PET (**a**) demonstrates subtle uptake in an enlarged right external node (**b**) which would be difficult to discern without knowledge of the CT findings. Correlation with prior contrast-enhanced CT (**c**) demonstrates the node has rim enhancement and central necrosis consistent with malignant aetiology. The rim of viable tumour is thin and below the resolution of PET imaging explaining the absence of significant uptake. Integration of CT morphology is critical in this case for accurate interpretation

The intensity of uptake in metastases usually parallels that in the primary site of disease. If not, another aetiology should be considered. For example, discordant low-grade activity in an enlarged lymph node in the setting of intense uptake in the primary tumour suggests it is unlikely malignant and more likely inflammatory or reactive. By CT criteria the enlarged node is ‘pathologic’ but the discordantly low metabolic signature further characterises this is as non-malignant since such a node is not subject to partial volume effects and therefore the intensity of uptake should be similar to the primary site. The exception is when the lymph node is centrally necrotic as a small rim of viable tumour is subject to partial volume effects with expectant lower intensity of uptake; integrating the CT morphology is therefore critical to reaching an accurate interpretation (see Fig. 9). Small nodes that are visualised on PET are conversely much more likely to be metastatic as such nodes are subject to partial volume effects.

The exception to this rule is tumours with a propensity for tumour heterogeneity at different sites. In follicular lymphoma or chronic lymphocytic leukaemia, discordant sites of high metabolic activity can be a specific finding for transformed disease. In malignancies with a range of well- to poorly-differentiated phenotypes (particularly endocrine tumours), it is possible to visualise tumour heterogeneity with different grades of disease at varying sites. The combination of FDG and a more specific tracer, which visualises the well-differentiated disease can be very useful to characterise this phenomenon, e.g. radio-iodine imaging for thyroid cancer or somatostatin receptor imaging for neuroendocrine tumours [6].

### Move beyond lesion counting and size measurement to lesion characterisation

The classical PET/CT indications involve primary staging, therapeutic monitoring, detection of recurrence disease or surveillance. The ability to non-invasively measure glycolytic activity, defining what we refer to as the “metabolic signature”, however, is a key feature of FDG PET/CT that is overlooked by many reporters. For the majority of malignant processes, the intensity of metabolic abnormality correlates with degree of aggressiveness or proliferative rate. For a metastatic malignant process that demonstrates no or minimal metabolic abnormality, this is usually a marker of low proliferative rate and indolent phenotype. Applying conventional diagnostic imaging paradigms, a negative PET/CT study in a patient with biopsy proven malignancy would be considered false-negative. A more useful report, however, would highlight the powerful prognostic information this provides. Providing such prognostic information was formerly the domain of pathology; a report which ignores the intensity of metabolic abnormality is missing a key utility of FDG PET/CT. Descriptively, we define SUV < 5 as “low intensity”, 5–10 as “moderate”, 10–15 as “intense” and >15 as “very intense”. Documenting the actual SUV in the report can be useful to avoid ambiguity with qualitative statements that may be interpreted variably.

Evolving literature suggests that intensity of uptake is an independent prognostic factor and in some tumour subtypes superior to histopathologic characterisation. Tumours with low uptake and commensurate indolent phenotype may include papillary thyroid cancer, neuroendocrine tumours, clear cell renal carcinomas and breast carcinoma. Each of these, however, can also demonstrate high intensity uptake commensurate with their spectrum of well- to poorly-differentiated phenotype, with the more aggressive phenotypes demonstrating high intensity uptake commensurate with their higher proliferative rate. PET can be used to guide targeted biopsy of the most intense site of metabolic activity.

There are some important exceptions to this broad principle as detailed below:

#### FDG negative but aggressive malignancy

The vast majority of aggressive malignant processes use aerobic glycolysis to derive a substantial amount of their energy, converting glucose to lactate by denying pyruvate access to the tricarboxylic acid cycle. This is termed the Warburg effect [7]. There, however, are a significant minority of tumours that utilise substrates other glucose such as glutamine or fatty acids as a source of the carbon atoms required for growth and proliferation. These allow glucose to be diverted into the pentose phosphate shunt pathway. The utility of FDG PET is diminished in this setting. This includes a subset of diffuse gastric adenocarcinomas, signet cell colonic adenocarcinomas and some sarcomas, particularly liposarcoma. Histologically, these are characterised by tumours with high proliferative rate but minimal GLUT-1 expression. There may be a role for other radiotracers such as fluorothymidine (FLT) or amino acid substrates in this setting.

FDG PET/CT has a finite resolution. However, this continues to improve with each generation of PET technology. Apparent FDG uptake is reduced in small volume disease due to partial volume effects, and also in areas of subject to movement, mainly due to respiration. The apparent intensity of uptake in small pulmonary metastases will be reduced due to both these phenomena. New reconstruction algorithms such as point spread function modelling can significantly improve lesion contrast but may also significantly impact the SUV of small lesions. Attempts to harmonise the semi-quantitative analysis of PET data require methods to deal with differences introduced by reconstruction algorithms [8]. Reduction in activity owing to respiratory motion is most evident in the lung bases and also the dome of the liver. Acquiring images with respiratory gating can be useful [9] but with experience this can often be recognised visually. As previously alluded to, enlarged necrotic nodes with only a thin rim of tumour are also subject to significant partial volume effects and can thus appear FDG negative (Fig. 9). Similarly, some aggressive sarcomas or mucinous tumours can also appear PET negative when the signal from cancer cells is dominated by the low uptake in adjacent by extra-cellular matrix or mucin production.

#### Intense FDG uptake but indolent neoplasm

**Fig. 10:**
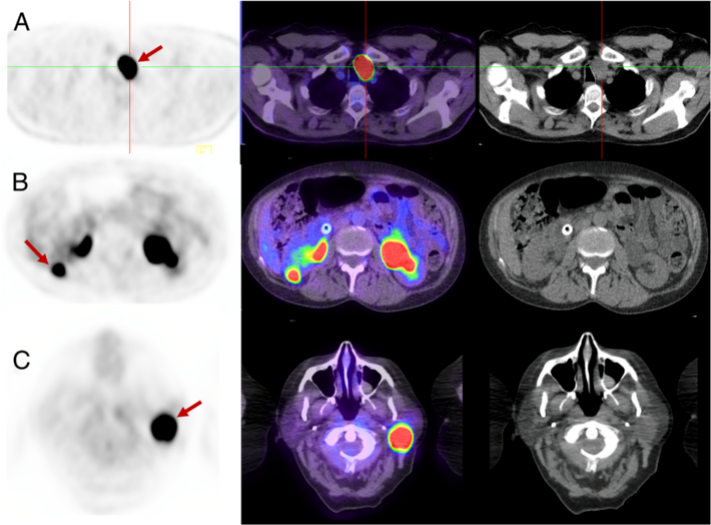
Three different patients with (**a**) Hurthle cell adenoma (thyroid oncocytoma), (**b**) renal oncocytoma and (**c**) Parotid Warthin’s tumour (parotid oncocytoma). Each has high SUVmax of 45, 22 and 35, respectively. In each case, the abnormality was present on imaging more than one year prior and unchanged in size. The very intense FDG uptake could be interpreted as suspicious for aggressive malignancy but the lack of temporal change was inconsistent with this. The lack of progression in a thyroid, renal or parotid lesion with very intense uptake is pathognomonic of benign oncocytomas

Some tumours harbour mutations that result in defective aerobic mitochondrial energy metabolism, effectively simulating the Warburg effect. Due to these mutations and consequent inefficient oxidative phosphorylation, a high amount of glucose is required for ATP production. Mutations in subunits of succinate dehydrogenase (e.g. *SDHB*) found in patients with hereditary paraganglioma and pheochromocytoma highlight this phenomenon. These have intense uptake on FDG PET/CT despite often having low proliferative rate. Benign oncocytomas, such as parotid, thyroid Hurthle cell or renal oncocytomas also harbour mutations of mitochrondrial oxidative phosphorylation resulting in high FDG activity (see Fig. 10). Uterine fibroids, hepatic adenomas, fibroadenomas of the breast and desmoid tumours are benign or relatively benign lesions that can have quite high FDG-avidity.

### Beware the staging scan which is actually a response assessment scan

Metabolic activity switches off rapidly following initiation of therapy. For example, following initiating of the tyrosine kinase inhibitor, imatinib, for treatment for gastrointestinal stromal tumours (GIST) metabolic activity changes from intense to negative within 24 hours. The same principle applies in a wide variety of circumstances so it is important to be aware whether or not the patient has commenced active therapy. Common examples where patients have commenced active therapy but the referrer is requesting “staging” includes hormonal therapy (eg. tamoxifen) in breast cancer, oral capecitabine in colorectal cancer or high dose steroids in Hodgkin’s lymphoma. In these setting, sites of disease may not be metabolically active confirming effectiveness of active therapy, but limiting the utility of PET to provide accurate staging. Accurate staging may not possible even shortly after treatment has commenced, a paradigm that is different from anatomic imaging where it takes some weeks for changes to occur. It is therefore critical to perform PET staging before commencement of anti-tumour therapy.

### Integration of CT data

Integrating the anatomic information provided by CT is important for accurate PET/CT interpretation as it may increase the specificity and sensitivity of PET findings. Detailed knowledge of the anatomic appearance of pathologic, inflammatory and benign processes is therefore critical to correctly interpret PET/CT. For example, focal intense abnormality on PET alone indicative of residual or recurrent lymphoma, may be revised to fat necrosis when CT appearances are integrated [10]. Likewise, lack of uptake in a lymph node may be revised from benign to malignant when the CT appearances of contrast enhancement rim enhancement and necrosis are integrated.

Many groups perform diagnostic CT studies with PET using a full-dose and contrast-enhanced acquisition including specialised regional protocols. In part, whether to perform this routinely depends on local practices, credentialing of reporting specialists, and reimbursement schemes. The potential advantage of routine diagnostic CT is improved anatomic localisation and definition, although we contend that low dose CT images reconstructed on modern generation devices usually provide sufficient detail with limited incremental value from “dedicated CT”. Moreover, patients have frequently already had a recent diagnostic CT, although this may diminish with increased utilisation of PET/CT as the first test rather than the last test. Without intravenous contrast, additional identification of typical oncologic complications such as pulmonary embolism or venous thrombosis cannot be identified. Nevertheless, if a “low dose CT” technique is utilised, it should not be considered “non-diagnostic” as it provides rich anatomic detail.

There are, however, situations where the acquisition of contrast-enhanced CT is preferred or can be tailored based on findings on the whole body low dose PET/CT without contrast in order to clarify the nature or anatomical relations of FDG-avid foci. Situations where we advocate full-dose, contrast-enhanced CT include localisation of cervical lymph nodes in head and neck cancer in the absence of systemic metastasis, especially to define necrotic nodes, the evaluation of liver metastases suitable for resection and for definition of pancreatic lesions [11]. In other cases, specific interventions, such as use of hyoscine and water to distend the stomach [12] or respiratory gating to resolve the nature of lesions that are subject to respiratory blurring [9], can further enhance diagnostic accuracy. The objective should always to utilise the complementary strengths of each modality to provide accurate diagnostic information pertinent to the individual patient’s care with the minimum risk and greatest convenience. Sometimes this will involve a dedicated and individualised CT acquisition protocol but for other patients, a non-contrast, low-dose protocol will be sufficient. Despite the logistic impost, our preference is to determine the need for and acquisition parameters for contrast-enhanced CT based on immediate review of the whole-body study without contrast and then doing a detailed loco-regional assessment as an additional acquisition, including pharmacological intervention if this may aid the diagnostic process.

When performing dedicated CT with higher dose and administration of intravenous/oral contrast may enable detection of abnormalities that are not FDG-avid, such as small hepatic or pulmonary lesions, many of these abnormalities are not malignant and represent incidental benign aetiology, thus potentially decreasing specificity. Just as integration of CT increases specificity of PET findings as discussed above, the converse can also be true. In malignancies which are known or expected to have high FDG uptake, we advise caution in reporting incidental findings on CT that are not FDG-avid as suspicious or malignant. Furthermore, equivocal abnormalities by CT criteria alone (e.g. an ovarian cyst) that would ordinarily mandate further investigation, may be characterised by the absence of FDG uptake as being extremely likely benign. The integration of PET to characterise incidental CT findings is important to decrease further investigations that may usually be mandated with CT alone. Over-sensitive reporting can lead to patient harm, or, worse still, might deny potentially curative treatment.

### Restaging studies

For oncologic FDG PET/CT, comparison with prior studies is critical to answer the clinical question. If the study is performed as an “interim” restaging study after commencement of therapy but before completion, in order to reach a valid or clinically useful conclusion findings must be interpreted in the context of known changes that occur at a specific timing and type of therapy. The most well studied use of interim PET is in Hodgkin’s lymphoma where repeat PET after two cycles of ABVD-chemotherapy provides powerful prognostic information and may improve outcomes by enabling early change of management. The use of interim FDG PET/CT is now a well established technique in high grade lymphoma with standardised reporting criteria [13].

In our experience, critical errors of interpretation can be made by comparison only with the prior study. For example, if PET/CT is performed too frequently, findings may be erroneously described as stable whereas comparison with the baseline study may clearly demonstrate regression or progression. Review of multiple serial MIP images over the course of therapies can enable rapid appreciation of changes not evident by comparison with the prior study. Knowledge of when treatment commenced is also critical for correct interpretation. For example, a restaging PET/CT performed 3 months after a baseline study demonstrating a “mixed response” with some lesions appearing larger and others smaller, could be better explained by progressive disease and subsequent response to therapy if it was known that therapy was only commenced 1 month prior to the restaging scan, with the initial scan therefore not representing a true baseline.

### Formulating reports

We aim to provide a succinct and structured report answering the clinical question under the following sub-headings:

- ▪ *Clinical notes:* The aim of this section is to identify the clinical question that needs to be addressed in the conclusion. Unfortunately, complete clinical information is frequently not provided by the referring physician, and therefore alternative sources of information must be sought including from the patient directly, via a patient questionnaire (see Table 2), electronic records or contacting the referrer.

**Table 2:** Our patient questionnaire that we use routinely to provide additional history that may assist PET interpretation

• When did you last eat? • Are you diabetic? If yes, what type of diabetic medications are you taking? When did you last have them? • Have you had any surgery, biopsies or day procedures in the last 5 years? Provide a brief list. • Do you have any prosthetic implants or drainage bags? • Have you had any recent infections? • List any other previous illness. • Do you suffer with any pain at the moment? If so where? • Have you ever had chemotherapy? When did you last receive this treatment? • Have you ever had radiotherapy? When did you last receive this treatment? • Are you taking any hormone therapy? • Are you currently takin any medicines or tablets? Please list. • Are you or were you ever a smoker? • For female patients, is there any possibility that you may be pregnant? When was your last menstrual period?
- ▪ *Technique:* We suggest including the following minimum details to document the method so that others can be reassured that the scan was technically adequate, and to enable similar acquisition parameters for subsequent scans: acquisition field-of-view, model of PET/CT scanner, reconstruction technique (e.g. use of time-of-flight), CT acquisition parameters (e.g. dose, use of contrast), FDG uptake time and blood glucose level.
- ▪ *Comparative studies:* Details of prior PET/CT and/or other imaging studies which have been directly compared.
- ▪ *Findings:* We divide this heading into *primary tumour (T), nodal metastases (N) and distant metastases (D)* sub-headings, followed by *other findings* to describe any incidental findings. For lymphoma we divide the report into *nodal* and *extra-nodal* sub-headings. We strongly prefer this to an anatomic report (e.g. head, neck, chest, abdomen/pelvis) as the important findings are documented first, and incidental findings last. The PET findings are presented first but are directly correlated with the associated correlative CT findings rather than performing sequential or separate PET and CT reports. An ideal descriptive report should enable the reader to visualise the findings even without having access to the images themselves. Where appropriate to support qualitative findings, specific measures including standardised uptake values (SUV), metabolic tumour volume and lesion dimensions should be included.
- ▪ *Conclusion:* This should provide a concise answer to the clinical question. We include the American Joint Committee on Cancer (AJCC) TNM stage for staging scans where our referral-base utilises this staging schema. For restaging, we summarise findings as a complete metabolic response, partial metabolic response, stable disease or progressive metabolic disease [14]. Where appropriate, especially when results are equivocal, we provide guidance to the referring clinician. To keep the report succinct, we avoid repetition of interpretative findings in *Findings* and descriptive findings in the *Conclusion*. Where a single unifying interpretation is not possible, we provide clinical useful differentials rather than an exhaustive list of all possibilities and try to indicate the most efficient means to address ongoing uncertainty, which might include suggesting an appropriate biopsy site or recommending further laboratory or imaging evaluations.

We include key images embedded in the report, consisting of serial MIP image demonstrating changes over time, and selected annotated fused PET/CT and CT images highlighting key abnormalities. Feedback from referrers indicates that integration of key images in reports is highly appreciated [15].

### Sensitivity versus specificity: what is optimal?

For cancer imaging with FDG PET/CT, we generally aim to report with high specificity acknowledging the consequent trade-off in sensitivity [16]. In our experience, high sensitivity reporting may lead to adverse patient outcomes by resulting in false positive findings and the potential to deny the patient curative-intent therapies, whilst also leading to a cycle of further investigations resulting in patient and physician anxiety. This approach is extended to incidental findings which are often clinically irrelevant in the context of patients with advanced malignancy.

### Conclusions

Correct and consistent thresholding of the PET window is essential for consistent and accurate interpretation. The PET coronal or cine MIP images provide the key information needed to obtain an overview that can often answer the clinical question. Not all metabolically active abnormalities are malignant and a variety of physiologic and inflammatory patterns must be recognised. Cohesive integration of functional and anatomic information provided by PET and CT, respectively, is essential for correct interpretation. In doing this, one must not merely use the PET to locate CT abnormalities which are then counted and measured. A key paradigm change with FDG PET/CT is its ability to non-invasively measure glycolytic metabolism, a hall-mark of aggressive malignancy. Integrating this “metabolic signature” into interpretation provides important information. Whilst the intensity of FDG uptake often correlates with disease aggressiveness, recognition of aggressive lesions that are not FDG-avid, and intensely FDG-avid but benign pathologies is essential.

## Abbreviations

FDG: ^18^F-fluorodeoxyglucose
MIP: Maximum intensity projection
SUV: Standardised uptake value
